# At the Crossroads of Clinical and Preclinical Research for Muscular Dystrophy—Are We Closer to Effective Treatment for Patients?

**DOI:** 10.3390/ijms19051490

**Published:** 2018-05-16

**Authors:** Kinga I. Gawlik

**Affiliations:** Department of Experimental Medical Science, Muscle Biology Unit, Lund University, Lund 221 84, Sweden; kinga.gawlik@med.lu.se; Tel.: +46-(0)46-222-0813

**Keywords:** muscular dystrophy, skeletal muscle, animal models, gene therapy, cell therapy, genome editing, clinical trials, extracellular matrix

## Abstract

Among diseases affecting skeletal muscle, muscular dystrophy is one of the most devastating and complex disorders. The term ‘muscular dystrophy’ refers to a heterogeneous group of genetic diseases associated with a primary muscle defect that leads to progressive muscle wasting and consequent loss of muscle function. Muscular dystrophies are accompanied by numerous clinical complications and abnormalities in other tissues that cause extreme discomfort in everyday life. The fact that muscular dystrophy often takes its toll on babies and small children, and that many patients die at a young age, adds to the cruel character of the disease. Clinicians all over the world are facing the same problem: they have no therapy to offer except for symptom-relieving interventions. Patients, their families, but also clinicians, are in urgent need of an effective cure. Despite advances in genetics, increased understanding of molecular mechanisms underlying muscle disease, despite a sweeping range of successful preclinical strategies and relative progress of their implementation in the clinic, therapy for patients is currently out of reach. Only a greater comprehension of disease mechanisms, new preclinical studies, development of novel technologies, and tight collaboration between scientists and physicians can help improve clinical treatment. Fortunately, inventiveness in research is rapidly extending the limits and setting new standards for treatment design. This review provides a synopsis of muscular dystrophy and considers the steps of preclinical and clinical research that are taking the muscular dystrophy community towards the fundamental goal of combating the traumatic disease.

## 1. Introduction

Skeletal muscle is the largest tissue in the human body, comprising approximately 40% of the total body mass. Striated muscle is perhaps the most structurally specialized among all organ systems. The unique subcellular architecture of the muscle enables it to empower body movement, but its function is more complex than that: skeletal muscle generates force that facilitates breathing and feeding, contributes to vision, is necessary for posture maintenance, and it also regulates body temperature, metabolism, and hormonal balance. Consequently, a muscle disease is detrimental to many aspects of human well-being. Diseases of striated musculature represent a major unmet medical need, significantly affect human mortality, involve a substantial proportion of patients with chronic conditions [1,2], and are associated with considerable economic and personal burden.

Muscle-affecting disorders that stem from direct abnormalities of the muscle tissue or the neuromuscular unit are called primary myogenic diseases, and include muscular dystrophies, myopathies (hereditary and acquired), myotonias, muscle spasms, sarcopenia (muscle atrophy in aging), metabolic disorders, and disturbances of neuromuscular transmission (e.g., myasthenia gravis). Muscle dysfunction can also be associated with diseases affecting other tissues (multiple sclerosis, amyotrophic lateral sclerosis, cachexia, spinal muscular atrophy, and peripheral neuropathies); these are called secondary muscle conditions [1]. In this article, I focus on muscular dystrophy (MD).

MD is considered the most devastating primary myogenic disorder, for several reasons: (1) it is caused by genetic defects that, to date, cannot be prevented; (2) the disease often manifests itself very early in life; (3) it leads to inevitable progressive muscle damage and loss of muscle function; (4) in consequence, patients either never learn to walk, lose ambulatory abilities , or have a very limited range of movements; (5) patients experience breathing difficulties, feeding complications, and often die in early decades of their life; and (6) the disease often causes severe defects in other tissues (central and peripheral nervous system, heart, eyes).

Over 50 MD forms and sub-forms, arising from mutations in numerous genes, have been identified to date. Molecular advances in the myology field have improved the diagnostic potential, increased our understanding of the disease pathogenesis, and facilitated treatment development, but current clinical management of MD still does not target the cause of the disease. Instead, management focuses on delaying the disease progress, providing relief from symptoms and facilitating everyday life. 

Successful design of clinical interventions for the disease has been limited due to several factors. Obstacles that impede advances of MD therapies include the size and complexity of the muscle tissue (high number of muscles involved), disease heterogeneity (mutations in different genes give rise to different phenotypes), and incomplete understanding of disease mechanisms (enormous intricacy of molecular interactions underlying the pathophysiology of each MD form). Many MDs are rare diseases, which also hinders the progress of treatment design, despite incentives to stimulate development of drugs for orphan diseases [3]. Nevertheless, linking preclinical knowledge with clinical experience is our only option in reaching the goal of successful clinical intervention for MD. 

In this review I describe the disease from both clinical and preclinical perspectives and focus on the most promising preclinical approaches that bridge the gap between basic science and potential MD treatment.

## 2. Muscular Dystrophy 

### 2.1. General Characterization

MDs are inherited disorders manifested by progressive muscle weakness, damage and wasting. They share several clinical characteristics, such as joint contractures, hypotonia, and myotonia. Muscle degeneration that stems from a genetic defect commonly leads to a drastic change in muscle morphology and, thereby, loss of muscle function. MD patients never achieve ambulation, lose ambulation, or have limited motor abilities. More than 50 MD types and subtypes have been described (mapped to over 40 genetic loci, Table 1) and that number is likely to increase due to rapid development of cutting-edge sequencing technologies. 

Although the basic clinical presentation of the disease is rather similar, a high degree of heterogeneity is a feature of MD: the severity, life expectancy, age of onset, progression, weakness and distribution (facial, axial, and appendicular musculature, proximal and distal muscle) vary considerably in different forms of the disorder (Table 2). This is due to mutations in the array of genes that give rise to different MD types (Table 1). Those genes encode for products that possess a broad range of biological functions (enzymes, signaling molecules, structural proteins, contractile unit proteins, and multifunctional proteins). Additionally, the exact roles of some of MD-afflicted proteins have not been fully characterized. The expression of MD causative gene products spans multiple cellular localizations: nucleus, nuclear membranes, cytoplasmic organelles (e.g., sarcoplasmic/endoplasmic reticulum, and mitochondrium), cytoplasm, sarcomere (muscle contractile unit), sarcolemma (muscle cell membrane), and extracellular matrix (Table 1). 

Some of the genes involved are also expressed in other tissues, resulting in clinical complications that contribute to the diversity of the disease. The complications associated with MD include cardiomyopathy, rigid spine (scoliosis, spine deformities), structural changes in the brain, peripheral neuropathy, eye defects, respiratory failure/difficulties, feeding difficulties, joint contractures, and bone fragility [4] (Table 2). Many MD patients have a considerably reduced life expectancy: death occurs in childhood, teens, or the third decade of life, and is usually caused by respiratory failure, severe infections of the respiratory tract, cardiac failure, or general distress that takes a toll on the whole-body function [4].

### 2.2. Classification and Frequency

MDs have been traditionally classified according to the clinical presentation: age of onset (e.g., congenital MD), progression, pattern of weakness distribution (e.g., limb-girdle MD), and mode of inheritance (X-linked or autosomal disorders). The development of cloning and genetic mapping a few decades ago enriched the classification and linked different conditions to distinct genetic defects. Continuous innovation of sequencing technologies shed even more light on the complexity of MD classification (Table 1). 

Mutations in different genes can give rise to the same clinical manifestation, and such cases are classified as the same disorder but divided into different sub-forms with their own OMIM numbers. This is often linked to defects in gene products that share the same cellular function (e.g., different glycosyltransferases in congenital MD or nuclear proteins in Emery-Dreifuss MD). Conversely, mutations in one gene could result in divergent phenotypes (e.g., fukutin-related protein is affected in limb-girdle MD or different types of congenital MD; lamin A/C mutations give rise to limb-girdle MD, Emery-Dreifuss MD, and congenital MD). Some of the genes related to MDs could also be a causative factor of conditions with no muscle involvement (e.g., lamin A/C) [4]. Mutation variants within the same gene contribute to the heterogeneity of the same MD type (e.g., different mutations in the laminin α2 chain gene are manifested with diverse phenotypes of patients with congenital MD type 1A (MDC1A)). 

Recently, a new classification for MDs related to glycosylation defects (dystroglycanopathies) has been proposed [6,7]. This group of diseases is now termed MDDG, with three subtypes: A (congenital MD with severe brain defects), B (congenital MD with milder brain defects), and C (limb-girdle variants, no brain defects) (see Table 1 and Table 2).

Most MDs are rather rare diseases, with the exception of the Duchenne variant, which belongs to the group of the most frequent genetic disorders (prevalence 6/100,000, incidence 1/3500–5000) [8,9,10]. Dystrophin deficiency accounts for over 80% of cases of MD worldwide [11]. The other most common MD forms include myotonic dystrophy (in the UK it is the most common muscle genetic disorder; [2]), fascioscapulohumeral MD, and Becker MD [12,13] (Table 1). Among congenital MDs, MDC1A (laminin deficiency), Ullrich syndrome (collagen VI deficiency), and dystroglycanopathies (MD with glycosylation defects) are the most frequent in European populations [14,15]. The disease occurrence often varies in different world regions, which is related to founder mutations—some disorders are more prevalent in Asian populations (Fukuyama congenital MD), whereas others prevail in European populations (Ullrich congenital MD, limb-girdle MD type 2A and 2I, MDC1A, tibial MD) (Table 1). It must, therefore, be considered that the prevalence can only be precisely assessed in given populations, and worldwide estimations are rough. Notably, only single cases have been described for some MD types (e.g., limb-girdle type 1H; Emery-Dreifuss 5, 6, 7; integrin α7 and α9 congenital MD, MDC1B), so the epidemiology parameters for these disorders cannot be precisely evaluated. Furthermore, all existing assessments may be inaccurate due to a lack of epidemiology data, unreported incidence, and imprecise diagnosis (especially in undeveloped regions of the world). Those numbers can change in the future and shift the epidemiology observations. 

### 2.3. Genetics

A wide spectrum of mutations has been reported, not only within the entire MD group of diseases, but also within single genes that give rise to a particular disease form. The list of new mutations and new case reports is constantly growing. Deletions, duplications, and point mutations (missense, nonsense, splice-sites, and premature stop codon mutations) affect the phenotype in different ways, depending on reading frame maintenance or loss (frameshift mutations). The mutations can lead to a complete deficiency of a gene product, its decreased expression, or the expression of an aberrant molecule, which could be linked to complete or partial loss of function. In general, in-frame mutations lead to a milder phenotype than frameshift mutations (e.g., Becker vs. Duchenne MD, respectively) [16]. It is noteworthy that defects in a protein that ultimately give rise to a dystrophic phenotype could be secondary (i.e., not directly linked to mutations in a gene encoding for that particular protein), but stem from mutations of another gene product that modifies various substrates to enable their function (e.g., mutations in glycosyltransferases that mediate glycosylation and, consequently, molecular interactions of dystroglycan).

### 2.4. Dystrophic Pattern of Muscle Biopsy

Muscle morphology is severely changed in all types of MDs, due to various defects within muscle cells and extensive damage of muscle fibers. Muscle degeneration is often a consequence of incapacity to withstand the mechanical stress, which leads to structural damage of various muscle compartments (e.g., sarcolemma tears, disrupted connection between the extracellular matrix and cytoskeleton, and sarcomere disruption), and subsequent muscle cell death (Table 1 and Table 2).

Importantly, muscle degeneration may not solely be caused by structural defects and decreased resistance to mechanical force. Some of the proteins implicated in MDs often play multiple roles in the maintenance of muscle physiology and function: they regulate signaling pathways, gene transcription, metabolism, protein degradation/turnover, cell survival, and substrate modification. Defects in those genes alter vital molecular processes, disrupt muscle homeostasis, and contribute to disease-specific abnormalities in the muscle ultrastructure.

**Figure 1:**
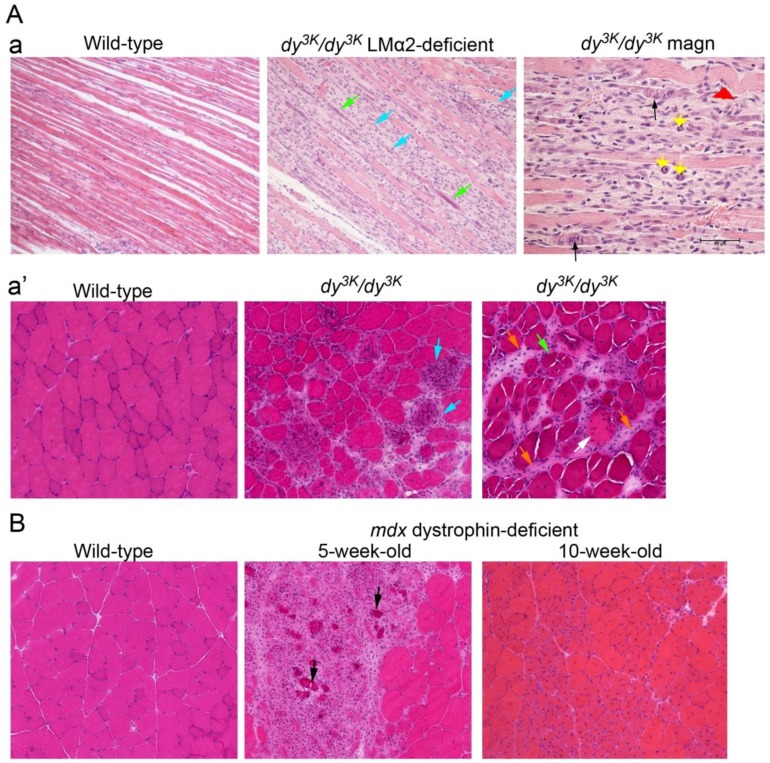
Common MD features on muscle biopsy. Muscles from animal models for MDC1A (**A**) and Duchenne MD (**B**) are shown. (**Aa**, **top panel**) Longitudinal sections of laminin α2 chain-deficient (LMα2) muscle from two-week-old *dy^3K^/dy^3K^* mice reveals a disruption of muscle fascicle: damaged and inflamed areas (blue arrows), small regenerating fibers with arrays of centrally-positioned nuclei (green arrows), dividing myoblasts (yellow arrowheads, magnified photo), regenerating fibers that are abnormal (undergo damage) (black arrows, magnified photo), and aberrant fibers (caterpillar shape, red arrowhead) are present. (**Aa’**, **bottom panel**) Muscle cross-sections from four-week-old mice display: fiber size variation, acute inflammatory response at the damaged fibers (blue arrows), degenerating/apoptotic/necrotic fibers (white arrow), regenerating fibers with centrally-located nuclei (green arrow), and fibrotic lesions (orange arrows). Normal (wild-type) muscles with tightly packed rectangular fibers are shown for comparison. (**B**) dystrophin-deficient muscles of *mdx* mice display a dramatic disruption of the muscle fascicle with focal necrosis, inflammation, and calcified fibers (black arrowheads) at five weeks of age. In 10-week-old muscle, active regeneration takes place (fibers with centrally-located nuclei), muscle regains fibers and its condition is not equally severe. Bar: 50 μm.

Despite the diversity of genetic defects involved in MD, the general characteristics of dystrophic muscle have been defined: atrophic fibers, variation in muscle fiber size, active regeneration cycles, the presence of necrotic/apoptotic fibers, fibrotic infiltrates, and muscle fiber loss (Figure 1) [1]. Features that might vary between biopsies obtained from patients suffering from different MD forms include: the degree, timing, and character of inflammatory response, sarcolemma damage, infiltration of adipose tissue, a change in the composition of fiber types (oxidative and glycolytic), the presence of ectopic calcifications, protein aggregates, vacuole, the proportion of apoptosis and necrosis, mitochondria abnormalities, nuclear abnormalities, and sarcomere disruption. Providing that muscle can regenerate the damaged fibers, it remains relatively functional, but those repaired fibers will never be as healthy as fibers undergoing regeneration under physiological conditions in unaffected individuals. It is inevitable that extensive muscle repair in MD is finally exhausted, muscle fibers are lost, and fibrotic lesions replace missing muscle cells. Fibrosis is often considered the ultimate step of the disease that triggers a loss of muscle function. Dystrophic features of muscle biopsy from various MD patients are presented in Table 2. 

### 2.5. Diagnosis

Although the overlap of MD symptoms complicates the diagnostic pathway, the increasing number and sophistication of the newest genetic and molecular technologies facilitate the diagnosis. However, evaluation of family history, basic physical investigation and symptom recognition (such as contractures, muscle stiffness, weakness, and atrophy) are important in determining the correct diagnosis and further diagnostic procedures. Common assessments include the distribution of weakness, blood tests (creatine kinase levels), muscle biopsy analysis, electromyography, muscle magnetic resonance imaging, neurological tests, heart tests (electrocardiogram), exercise assessment and, finally, genetic tests (screening for mutations in a predicted gene) [8]. As proper disease management already at the onset greatly delays disease progress [4], it is crucial to diagnose the disorder as early as possible. The mean age of diagnosis, even for common variants such as Duchenne MD, is delayed by about two years after the manifestation of the early clinical signs [17] because the first symptoms are often overlooked. Consequently, neonatal screening programs [4] and even prenatal testing have been recommended [18,19]. 

### 2.6. Current Management

Current management of MD patients does not offer a cure, and instead focuses on delaying the disease progression and relieving symptoms. However, constantly increasing knowledge about the disease types, their mechanisms, and complications have led to highly refined standards of care, better implementation of medical advances, more effective prevention of complications and, thereby, improvement in the clinical course, quality of life, and prolonged survival of patients [4]. 

The principles for treating individuals with various MDs are similar, but vary in gradation [20]. Management of complications includes physiotherapy, non-invasive ventilation support, manually- and mechanically-assisted coughing techniques, posture correcting surgeries, use of equipment that supports ambulation, maintains posture, and prevents contractures (braces, mobility aids, night splints), tube feeding, enterogastrostomy, and a proper diet rich in supplements [4,20,21]. The significance of physiotherapy is increasing, as exercise and stretching clearly minimize joint contractures and spinal deformity, strengthen bones, prolong ambulation, and maintain the best possible level of health and function [20]. 

The possibilities afforded by pharmacological treatment of MD have been rather narrow, mostly limited to glucocorticoids (anti-inflammatory agents) and drugs that target complications in different tissues (e.g., heart medication and anti-epileptic drugs). Anti-inflammatory steroids have been shown to improve muscle strength of Duchenne MD patients, but they do not prolong life expectancy. In addition, they have long-term side effects [8]. Deflazacort is currently the most widely used drug, as it prolongs ambulation and causes milder side effects [8,22]. 

In summary, a combination of management strategies is essential, as it is likely to yield a better outcome of patient condition. However, regardless of the medical interventions attempted to date, MD remains incurable and the disease progression is currently unavoidable. 

### 2.7. Animal Models

Animal models constitute the major preclinical tool in elucidating disease mechanisms and evaluating potential diagnostic and therapeutic approaches. Mouse models are available today for most types of MD: either spontaneous mutants exist, or a wide range of genetically-modified mice has been generated over recent decades, not only to mimic patients’ general phenotypes, but also to study the effect of specific mutations [23,24] (Table 3). New mouse models will continue to be developed because of pioneering methods, such as CRISPR/Cas9 technology (see Section 3.2.2). 

The availability of larger organisms (such as dogs) as the disease model is tremendously beneficial for studying potential treatment possibilities [25]. Dog models are used in research of Duchenne MD (golden retriever muscular dystrophy dog) [24], but the demand for larger organisms in muscle disease research is far from satisfied. 

Finally, the use of smaller organisms, such as zebrafish, is also of great help in preclinical studies and should not be underestimated. They provide low cost and short-term read-out opportunities in research, especially in sophisticated mutagenesis and in large-scale genetic and therapeutic screening [26].

*Mdx* dystrophin-deficient mouse (point mutation in exon 23) is the most widely used mouse model in preclinical studies of MD [27,28]. Many treatment approaches tested on *mdx* mice led to the improvement of their phenotype (reviewed in [3,29]) and set trends for therapy concepts. Although this mouse has proved to be a valuable model, its general condition and the muscle wasting phenotype present themselves in a much milder form than in humans [23], so studies in more severely-affected mouse strains are crucial. For example, *mdx/utrn* mouse, lacking both dystrophin and its homologue utrophin, is a more adequate mouse model for Duchenne MD [30,31]. Additionally, the exon 23 point mutation in the dystrophin gene on different genetic backgrounds (different mouse strains) results in a variable degree of phenotype severity [32]. Table 3 shows a summary of animal models for MD. 

## 3. Preclinical Studies: Strategies for Treatment 

The growing understanding of genetic and molecular mechanisms of MD pathology has provided new clues for treatment and has led to innovative therapy approaches in preclinical research. State-of-the-art treatment methods are continuously evolving. 

Historically, two major concepts for curing genetic disorders have been established: reversing a primary defect (restoring the original function of a protein) or targeting secondary disease outcomes. Numerous approaches for rescuing primary defects have been developed in recent decades: gene therapy (delivery of the non-mutated gene or a paralogous gene), stem cell therapy, protein therapy, and mutation repair strategies. Based on studies in animal models, it has become clear that reversing the primary defect would almost certainly be more beneficial for the condition of individuals with MD [3,33,34]. This concept is much easier to implement in mice, whereas various gene manipulation techniques are either not applicable in humans or the gene restoration in patients has encountered obstacles (e.g., inefficient gene delivery to multiple muscles, low expression levels, immune response towards new antigens, alternations of patients’ genome, toxicity). On the other hand, since MD pathology is extremely complex, targeting secondary defects of the disorder would probably require modification of multiple cellular processes (e.g., boosting regeneration, inhibiting cell death and fibrosis, modulating inflammation, metabolism, and protein turnover). The current consensus is that combination therapy targeting both the primary and secondary defects of the disease is probably needed, and this has been confirmed in preclinical studies in mice [35,36]. 

Much of the research regarding treatment has focused on Duchenne MD, because of its severity and relatively high frequency. For that reason, most of the preclinical studies that hold promise and that I chose to describe below are performed in the *mdx* mouse model. However, I will also focus on successful approaches for less frequent MDs for two reasons: (1) *mdx* mice display a much milder phenotype than Duchenne MD patients, so prospective results in dystrophin-deficient mice are often negatively verified in early phases of clinical trials. Mouse models for other types of MDs most often mirror the human condition more adequately; and (2) I would like to pay more attention to research on rare muscle diseases. 

Many approaches that have been tested on animals to date preclude their complete description in the context of this review. Therefore, I have chosen the most novel and promising strategies together with the studies that illustrate the current directions of preclinical research in the myology field. I have also focused on MDs involving genes for extracellular matrix and cell adhesion complexes (dystrophin-glycoprotein complex).

### 3.1. Rescue of the Primary Genetic Defect: Classical Concept

**Figure 2:**
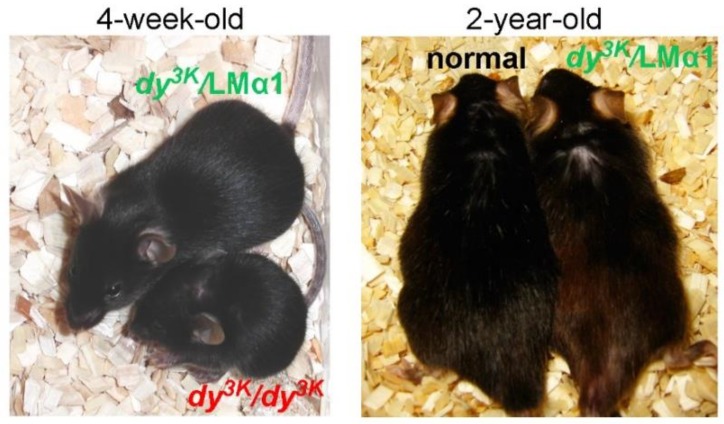
An example of a rescue of dystrophic phenotype in a mouse model for congenital MD. The mouse lacking laminin α2 chain (*dy^3K^/dy^3K^* in the **left picture**) at the terminal stage of the disease shows severe muscle wasting and severe overall phenotype. The laminin α2 chain-deficient littermate overexpressing laminin α1 chain (*dy^3K^*/LMα1) displays significant improvement of multiple aspects of the disease. The laminin α1 transgene prevents MD throughout life (**right picture**): two-year-old *dy^3K^*/LMα1 and normal littermate mouse present similar outward features.

Twenty-five years have passed since transgenic expression of the full-length dystrophin gene in *mdx* mice proved the concept of gene therapy for MD [37]. Since then, various modifications of the classical transgenic approach have been tested in mice, to bypass the unfeasibility of transgenic strategies in humans and to circumvent gene therapy hurdles. For example, mini-dystrophin genes were designed [38,39] to accommodate the limited cloning capacity of viral vectors (which became an extensively explored gene therapy tool). Additionally, to avoid inflammatory response towards new antigens, ‘surrogate’ homologous genes were overexpressed in dystrophic muscle to restore biological function of missing/abnormal protein. The most spectacular examples of such an approach include utrophin upregulation in *mdx* mice and laminin α1 chain overexpression in laminin α2 chain-deficient mice, both of which rescued the dystrophic phenotypes of respective dystrophic models [40,41,42,43] (Figure 2, Video 1). 

These relatively simple paralogous gene therapy approaches could be an effective weapon against dystrophin and laminin gene defects, as they aim at hitting broad mutation spectra and, in principle, are suitable for curing patients with all dystrophin and laminin mutations, respectively. Analogically, this strategy could work for patients with other gene defects if an appropriate paralogous gene exists. For example, the putative glycosyltransferase LARGE has been shown to functionally bypass the α-dystroglycan glycosylation defects caused by mutations in genes for distinct glycosyltransferases [44,45]. 

In 2001, Moll and colleagues pushed forward the concept of non-homologous repair of the primary defect. They engineered the mini-agrin protein that restored the link between extracellular matrix and transmembrane receptors in laminin α2 chain-deficient dystrophic muscle [46], which was sufficient to rescue the dystrophic symptoms. These results demonstrated that a clever molecular manipulation can serve as a paradigm to create therapeutic tools restoring muscle function in MD patients. 

This led to other inspiring and successful attempts using engineered molecules. For example, laminin/nidogen chimeric protein was designed to strengthen the connection between the truncated form of laminin and other components of specialized extracellular matrices (basement membranes) in congenital MD type 1A (MDC1A). This aberrant laminin molecule lacking the N-terminal domain is unable to polymerize and form a basic frame of basement membranes. Approximately 20% of patients with laminin α2 chain mutations suffer from a similar molecular defect [15,47], and the *dy^2J^/dy^2J^* mouse mirrors this deficiency. The nidogen molecule provides a link between basement membrane modules and the expression of the chimeric protein in *dy^2J^/dy^2J^* dystrophic muscle re-established laminin polymerization, restored basement membranes, and ameliorated the phenotype of *dy^2J^/dy^2J^* animals [48]. Importantly, combinatorial expression of laminin/nidogen chimeric protein and mini-agrin (currently termed linker proteins) in a more severe mouse model for laminin deficiency (*dy^w^/dy^w^* mice displaying almost complete deficiency of laminin α2) fully recovered basement membrane and rescued the muscle phenotype, establishing an even stronger basis for potential treatment with engineered molecules [49]. 

Nevertheless, approaches aimed at targeting primary defect repair need to find a common denominator: effective transition from preclinical to translational research. The leading concepts are presented below. 

#### 3.1.1. Virus Delivery: From Proof of Concept to Implementation

The gene therapy concept was developed as early as the 1970s, yet there are no FDA-approved gene therapy products and, in Europe, only one product has been approved so far (for lipoprotein lipase-deficiency) [3]. Adeno-associated viruses (AAV) are the most promising gene delivery vehicles for treating dystrophic muscles, due to low pathogenicity, the ability to infect non-dividing cells, reasonable packaging capacity (app. 5 kb), and effectiveness in transducing skeletal muscles after both intramuscular and intravenous injections [50,51,52]. 

Virus-driven delivery of therapeutic genes has been successful in various animal models for MD. An AAV-mediated intramuscular gene transfer of sarcoglycan genes into mice suffering from deficiencies of various sarcoglycans (α-, β-, δ-, γ-sarcoglycan) resulted in substantial recovery of each sarcoglycan molecule expression and significant improvement of the muscle phenotype in the respective mouse models [53,54,55]. Both intramuscular and intravenous injections of AAVs carrying the FKRP gene were also successful in a FKRP knock-in mouse model for the LGMD2I missense mutation [56]. Similarly, LARGE-AAV gene transfer rescued glycosylation defects in LARGE and POMGnT1 mice [45].

The full-length laminin gene is too large for packaging into an AAV vector, but the mini-agrin transgene that functionally replaced the laminin α2 chain in dystrophic muscle (see above, [46]) was successfully used in somatic gene therapy to treat congenital MD type 1A (MDC1A) in two different mouse models (intramuscular and intraperitoneal injections) [57]. Similarly, systemic administration of AAV pseudotype 6 achieved widespread transduction of shorter dystrophin forms (mini-dystrophin) in both cardiac and skeletal muscles in *mdx* mice [51]. Importantly, this approach was also successful in dystrophin-deficient dogs [58,59,60]. Long-term maintenance of mini-dystrophin expression in the dystrophic dog (a large organism that also represents the relevant animal model for Duchenne MD) sets the stage for clinical trials in human patients. One trial using AAV-mini-dystrophin delivery is currently in progress (NCT02376816). Due to promising results in mice and non-human primates, AAV therapy will also be tested in patients with limb-girdle MD type 2D, 2B, 2I, and FKRP-deficient congenital MD (reviewed in: [3,61]). Consequently, the gene therapy approach using viral vectors shows renewed optimism [61], and there are possibilities to expand the utility of AAV, especially in the context of combining gene therapy ex vivo with new advances in the cell therapy field [50].

#### 3.1.2. Cell Therapy

Evidence has been accumulating for the stem cell dysfunction in MD [62], which eventually leads to inefficient muscle regeneration and contributes to loss of muscle function. New regenerative medicine-based therapies for skeletal muscle using human pluripotent cells are needed and are currently being widely explored [62]. Cell therapy can be utilized for cell replacement, offering the potential to reverse muscle atrophy, or could be used for the correction of the primary genetic defect. 

Both autologous and allogeneic cell transplants are considered. Numerous cell types have shown myogenic potential and have been tested for restoration of muscle function in dystrophic animal models. These cells include muscle satellite cells, muscle-derived stem cells (mesenchymal-like stem cells residing in muscle), but also cells derived from bone marrow, vessel wall (mesoangioblasts, pericytes), and dermis (reviewed in [63]). Mesoangioblasts transplanted into dogs with dystrophin deficiency have shown relatively high efficacy of dystrophin expression restoration [64]. These cells have been tested in numerous mouse models [65,66] and were considered to be one of the most successful cell therapy tool for treating MD. Despite undisputed potential of cell therapy, difficulties with cell isolation, expansion, efficient delivery and engraftment, cell survival, and stable expression of a therapeutic protein hinder its use in clinics. Nevertheless, autologous cell therapies with mesoangioblasts, myoblasts, bone marrow cells, and mesenchymal cells are currently in various phases of clinical trials for Duchenne/Becker, oculopharyngeal, and fascioscapulohumeral MD [67,68].

#### 3.1.3. Protein Therapy

Protein therapy is based on delivery of a therapeutic protein into diseased muscle. The simplicity of the concept and escaping involvement of genetic material and gene expression-related steps are, without a doubt, advantageous and unique among currently-proposed methods. The assumptions behind protein therapy could seem ideal for quick implementation, but the approach remains controversial. The stability of therapeutic proteins and long-term effect of protein therapy are the issues that have been questioned. The protein delivery into diseased muscle is as tricky as gene delivery, and the immune response towards a foreign antigen is not eliminated when the administered therapeutic agent resembles, or is identical, to a missing protein. 

Several studies in mice have now confirmed that injected protein (intramuscular, intraperitoneal and intravenous injections) can be incorporated into muscle at sufficient levels to mediate the phenotype correction of dystrophic mice (mainly dystrophin-deficient *mdx* mice, but also laminin α2 chain-deficient mice). The proteins delivered so far into dystrophic mice (micro-utrophin, laminin-111, biglycan, wnt7, and galectin-1) either targeted the primary defect [69,70] or influenced biological processes that led to improved structural stability and enhanced regeneration of muscle tissue [71,72,73,74]. It is noteworthy that the severe condition of laminin α2 chain-deficient *dy^w^/dy^w^* dystrophic mice (mouse model for MDC1A) was only partially ameliorated with this approach [69], despite targeting the primary defect (replacement of laminin-211 with laminin-111). In contrast, similar functional replacement by transgenic means showed the remarkable rescue of the phenotype in a more severe mouse model for MDC1A (*dy^3K^/dy^3K^*) [42,75]. An ultimate challenge for protein therapy will be large-scale production of human recombinant proteins for therapeutic injections. 

#### 3.1.4. Endogenous Up-Regulation of Paralogous Genes

Triggering muscle-specific endogenous upregulation of homologous/therapeutic protein that is already expressed in a patient body (either in low levels in muscle or in other tissues) is a molecular manipulation that has not yet been fully explored. This strategy is extremely promising and could become a leading therapy for MD. Increasing the expression of a gene could be achieved via small-compound drugs that interact with promotor sequences of a gene of interest and specifically trigger the transcription machinery. It involves drug discovery or, preferably, could be achieved by screening compounds that are already approved for other indications. 

The utilization of endogenous target genes provides an elegant solution to various problems arising from the gene therapy strategies and solves the issue of immune response towards foreign antigens. The use of small artificial molecules with favorable absorption, distribution, and metabolism also offers clear advantages in terms of delivery, stability, and availability [76]. Utrophin expression has been shown to be upregulated through activating its promotor by pharmacological compound GW501515, nabumetone, or artificial transcriptional regulator Jazz [76,77,78], and this upregulation rescued the phenotype of *mdx* mice [77,78]. Utrophin expression has also been shown to be regulated by post-translational mechanisms [79] and it is possible that combining drugs that act at both transcription and translation levels would result in the best outcome. 

We have just begun to understand the complexity of translation regulatory mechanisms (e.g., micro-RNA-related mechanisms). In summary, further studies are necessary to discover new candidate compounds or to implement new technologies (for example CRISPR/Cas9) and to test an endogenous upregulation approach in animal models for different MDs. For example, upregulating laminin α1 chain in MDC1A could be an obvious choice for such a strategy, since laminin α1 chain expression in laminin α2 chain-deficient dystrophic muscle greatly improves the phenotype [42,43,75] and increased expression of laminin α1 chain in vitro and in vivo using the CRISPR/Cas9 system has been recently achieved [80]. 

### 3.2. Targeting Primary Genetic Defects: Mutation Repair

Rapid development and significant improvement of genomics technologies opened new avenues for targeting the primary genetic defect through genome editing, which became an alternative to the classical gene therapy approach. The possibility to correct mutations in somatic cells is equally challenging, yet could be the future for therapy of muscle disorders. 

#### 3.2.1. Exon Skipping

Exon skipping is aimed at reframing the disrupted transcripts using antisense oligonucleotides as a tool for the ‘excision’ of a mutated exon. Antisense-mediated modulation of splicing results in an expression of truncated, but functional, protein. Not all mutations are suitable for exon skipping, but certain mutations in the dystrophin gene (e.g., in exon 51, approximately 13% of patients) are ideal for this approach, since the affected exon designated for skipping does not carry an essential function. Numerous studies have been carried out to drive exon skipping in mice and dogs [3,81,82,83]. Two types of antisense oligonucleotides have been tested in clinical trials (2′-O-methyl phosphorothioate (PEO051) and phosphorodiamidate morpholino oligomer (PMO AVI-4658, eteplirsen)) [84,85,86,87,88], but have failed to show discernible clinical benefit, probably due to inadequate rescue of dystrophin expression [89]. 

Injections of ‘naked’ oligonucleotides may lie behind the inefficiency of dystrophin restoration. Nevertheless, after a controversial debate surrounding the efficacy of AVI-4658 (eteplirsen), it received accelerated approval from the US Food and Drug administration in late 2016 (Sarepta Therapeutics Inc. Eteplirsen briefing document (NDA 206488, http://www.fda.gov), making it the first, and currently the only, FDA-approved drug for Duchenne MD. This approval is encouraging, but we must wait for the long-term verification of patients’ responsiveness and, currently, the confirmatory phase III trial is ongoing to secure final approval from the FDA [90]. 

The exon skipping avenue is constantly explored. Viral delivery of oligonucleotides in a canine model for Duchenne MD resulted in high levels of dystrophin expression and was safe for dogs [91]. An AAV-mediated delivery of oligonucleotides may lead to more efficient genome editing.

Recently, a new class of antisense oligonucleotides (tcDNA) has been designed that have shown tremendous pharmacological properties and unprecedented uptake by many tissues, as tested in *mdx* and *mdx/utrn* double-knockout mice (these mice display a much more severe phenotype and better resemblance of human condition) [92]. tcDNA particles display increased affinity to mRNA, increased nuclease resistance, and spontaneous self-association that features modern nanoparticle delivery systems. 

Taken together, these results refresh the concept of clinical trials for exon skipping. 

#### 3.2.2. CRISPR/Cas9 Genome Editing 

CRISPR/Cas9 is a breakthrough technique that holds enormous promise for the treatment of various genetic diseases, and has the potential to replace the classical gene therapy approach, which so far has not lived up to its expectations. The brilliance and innovation behind the CRISPR/Cas9 system is a light in the tunnel for patients and brings excitement into the scientific community. This technique offers efficient gene repair solutions and has a capacity to target a wide range of mutations. More than 2000 articles on CRISPR/Cas9 have been published since the original method was described in 2013 [93], and the system is now commonly used in preclinical studies. 

Briefly, single-guide RNA (sgRNA) guides Cas9 endonuclease into a specific site, where it generates double-strand breaks. DNA repair then takes place (non-homologous end joining (NHEJ)), or an exogenous template provides homology-directed repair (HDR), to precisely modify the genome at a target locus. In the field of MD, CRISPR/Cas9-mediated genome editing was used for the first time to correct the dystrophin gene mutation in the germ line of *mdx* mice, which carry a nonsense mutation in exon 23. This intervention in cells that actively divide resulted in a complete prevention of the dystrophic phenotype, with allele restoration ranging between 43% and 81% [94]. 

So far, zygote manipulation is not feasible in humans, so AAVs were used to deliver CRISPR/Cas9 machinery into postnatal somatic cells of *mdx* mice. Three independent studies have shown that postnatal deletion of exon 23 resulted in restored expression of truncated dystrophin and significant recovery of dystrophin function, followed by improvement of the *mdx* phenotype [95,96,97]. 

What is even more encouraging is that CRISPR/Cas9 technology has shown flexibility for a broader range of dystrophin mutations. *Mdx^4cv^* mouse mutant harboring a nonsense mutation within exon 53 (corresponding to mutations carried by a large population of Duchenne muscular dystrophy patients) has been subjected to AAV delivery of distinct CRISPR/Cas9 constructs in order to achieve excision of defective exon (NHEJ) within an open reading frame or to repair mutation directly, which required successful utilization of HDR [98]. Both approaches resulted in widespread expression of dystrophin in muscle and heart and showed potential applicability to different mutational contexts: mutations in exons encoding non-essential or essential domains of dystrophin, respectively [98]. The CRISPR/Cas9 approach has also been shown to correct the pathogenic splice-site defect in the laminin α2 chain gene in the *dy^2J^/dy^2J^* mouse model [99], and approximately 20% of individuals with MDC1A carry mutations in *LAMA2* splice sites [15,47]. 

In summary, CRISPR/Cas9 genome editing has passed multiple proof-of-principle tests and has demonstrated a strong arsenal against neuromuscular disorders. Numerous fine-tuning improvements are under development, such as elimination of off-target DNA cutting. Nevertheless, effective delivery of CRISPR/Cas9 constructs and achieving high efficiency of genome editingin human patients face the same types of obstacles as the delivery of vectors in the classical gene therapy approach. Will the CRISPR/Cas9 technique live up to expectations? 

#### 3.2.3. Alternative Use of Antisense Methods

Genomic expansions of simple tandem repeats give rise to toxic RNAs in myotonic MD. The use of a morpholino antisense oligonucleotide CAG25 in a mouse model for myotonic MD (*HSA*^LR^ mice with multiple CUG repeats) has been shown to inhibit deleterious interactions of proteins with pathogenic RNAs and reduce its overall burden [100]. Antisense oligonucleotide technology can, therefore, be designed for genome editing in various types of mutations. 

#### 3.2.4. Suppression of Stop Codons

Nonsense mutations that give rise to in-frame stop codons in messenger RNA coding regions can be pharmacologically targeted. Drug-induced translational read-through of the premature codon stop enables the expression of full-length functional protein. This therapeutic strategy applies to approximately 15% of Duchenne MD patients who have nonsense mutations [101]. Ataluren (PTC124) suppressed the nonsense mutation in *mdx* mice [102], showed no toxic effect, and promoted mild dystrophin expression in patients recruited for early phases of clinical trials [103,104]. However, the phase 2b clinical study revealed only marginal functional benefit in a 6-min walk test and did not include dystrophin protein expression data [105,106,107,108,109]. Nonetheless, patients voiced positive effects for their well-being and a bell-shaped dose response curve was achieved [110], moving PTC124 into phase 3 clinical trials (NCT01826487). This large study was finalized recently, showing mild positive effect on a certain group of patients [111]. The first phase 2b/3 trials carried out in Duchenne MD and conditional global approval for ataluren in Europe (http://www.ema.europa.eu) have become a milestone in the development of a potential therapy for MD patients [112], but the FDA has not approved the drug due to inconclusive data. More pharmacokinetic and preclinical studies of drugs targeting the stop codons are needed to further pursue this line of treatment. 

### 3.3. Targeting Secondary Defects of Muscular Dystrophy

The complicated character of dystrophic disorders is largely dictated by secondary pathologies that result from a primary genetic defect. These pathologies act in concert, causing a domino-like effect. As a result, they severely exacerbate the dystrophic phenotype, making the disease difficult to target comprehensively. This has led to a focus on reversing the secondary outcomes of MD. This tactic has become an attractive alternative to the complexity of genetic manipulations. Even if a primary defect is still present, prevention of deteriorating processes (e.g., inflammation, fibrosis, cell death, muscle repair insufficiency) could lead to partial restoration of muscle function. One advantage of such approaches is that approved drugs for other diseases often fit the strategy for curing MD (e.g., anti-fibrotic and anti-inflammatory drugs, proteasome inhibitors, anti-diabetic compounds, blood pressure drugs, and immunosuppressants; see below). Many downstream pathologies have been successfully inhibited with pharmacological approaches (but also transgenic strategies) in animal models for MD. Consequently, strategies for targeting the secondary disease mechanisms are multi-dimensional and this bodes well for future therapies, especially that combinatorial treatment could be required for the optimal outcome. 

Since dystrophic muscles in most MD cases are hampered with muscle cell death, impaired regeneration, and increased fibrosis, inhibition of these processes has attracted broad interest. Targeting necrosis has been efficient in *mdx* mice, but also in δ-sarcoglycan knockout mice, through modulation of pathogenic mechanisms (mitochondrial dysfunction, oxidative stress, and blood flow impairment) using various pharmacological compounds [113,114,115,116,117] (reviewed in [29]). Apoptosis, on the other hand, is a hallmark of MDC1A and treatment with anti-apoptotic agents omigapil and doxycycline assuaged muscle pathology in laminin α2 chain-deficient mice [118,119]. It is noteworthy that omigapil has now entered clinical trials for congenital MD patients (NCT01805024). Apoptosis has also been indirectly targeted in collagen VI-deficient mice by counteracting mitochondrial permeability with cyclosporin A (an immunosuppressant) or Debio-025 (initially developed for the treatment of hepatitis C) [120,121]. Combating mitochondrial pathogenesis in these mice had a positive impact on other MD-related defects, such as muscle degeneration and ultrastructural lesions of sarcoplasmic reticulum [120,121,122]. 

It has become even more evident that the secondary abnormalities meet at the crossroads of muscle pathology, so triggering one of de-regulated processes often positively affects the other. For instance, administration of a TGF-β-blocking reagent losartan (which is approved for hypertension prevention in humans) in *mdx* mice targeted the pro-fibrotic pathway and resulted in normalized muscle architecture and increased muscle repair [123]. Similarly, an inhibitor of Smad3 phosphorylation downstream of TGFβ signaling (halofuginone) decreased the activation of fibroblasts in MDs with fibrotic presentation (dystrophin and laminin-deficiency) [124,125], but also improved the condition of dysferlin-deficient dystrophic mice with minor fibrosis involvement, probably due to a direct effect of halofuginone on muscle regeneration [126]. 

Losartan has also been shown to act synergistically with muscle regeneration-stimulating hormone IGF or growth hormone (a readily-available growth-promoting drug that is safe for children) in laminin α2 chain-deficiency [127]. Likewise, anti-apoptotic treatment together with administration of recombinant IGF-1 enhanced the improvement of dystrophic phenotype of laminin α2 chain-deficient mice [128]. Losartan has now been tested in clinical trials for the treatment of cardiomyopathy of Duchenne MD patients [129] (NCT01982695) and has been a therapeutic candidate for trials in patients with MDC1A [130]. 

The significance of finely-tuned changes of an inflammatory and fibrotic milieu driven by a pharmacological agent has been recently demonstrated in a study with a tyrosine kinase inhibitor nilotinib, which is approved for treatment of myelogenous leukemia. Nilotinib timed the transition between TNF and TGF-β-expressing macrophages and promoted apoptosis of pro-fibrotic fibro/adipogenic progenitors in dystrophic muscle of *mdx* mice [131]. Such a molecular shift lessened a few aspects of muscle pathology and, hopefully, nilotinib could also be effective in MD patients. Lessening inflammation has been particularly efficient when blocking the P2RX7 “danger” receptor that recruits inflammatory cells into dystrophic muscle [132,133]. Notably, macrophage polarization and its impact on muscle regeneration have only recently been unveiled [134,135] and our understanding of detailed inflammatory processes could be the foundation of future therapies for inflammation-afflicted MDs. 

Other downstream pathologies related to MD, such as imbalanced protein turnover, have also been targeted in relevant mouse models. Enhanced proteasomal degradation has been found to be a hallmark of dystrophin and laminin-deficient MD [136,137], and its inhibition with bortezomib could feature prevention of muscle atrophy and fibrosis [138,139]. Autophagy is one of the systems implicated in degradation of proteins and organelles, and its activity has also been shown to be either attenuated or increased in dystrophin, collagen VI, laminin α2 chain and lamin-deficiencies [140,141,142,143]. Autophagy-related treatments have been successfully explored in mouse models for these diseases, with use of genetic and pharmacological approaches, or through application of a low-protein diet [140,141,142,143]. Autophagy inhibition in laminin α2 chain-deficient mouse (with 3-methyladenine) or autophagy boost in *mdx* and collagen VI-deficient mice led to normalization of muscle morphology and function [140,141,142]. Autophagy activation with low-protein diet is now being tested in Ullrich congenital MD patients [144]. Lamin-deficient MDs display altered protein balance machinery due to increased mTORC1 signaling, and pharmacologic reversal of elevated mTORC1 by rapamycin has effectively improved skeletal and cardiac muscle function in lamin A-deficient mice. Rapamycin administration has also improved autophagic-mediated degradation in these animals [143]. Since skeletal muscle acts like an endocrine organ and has a tremendous impact on whole-body metabolism, it is not excluded that modulating muscle metabolic machinery through master growth regulators (such as mTORC1) that sense and integrate diverse nutritional and environmental cues, could bring an important aspect to treatment of MD patients [145]. Consequently, there is growing evidence that muscle metabolic processes are drastically altered in MD [146,147,148]. 

Not only pharmacological compounds, but also the use of antibodies that target specific signaling pathways regulating biological processes mentioned above (fibrosis, regeneration), has generated encouraging results in preclinical studies. For example, inhibition of lysyl oxidase-like-2 (involved in collagen synthesis) or connective tissue growth factor (promoting fibrosis) with specific antibodies has been shown to be beneficial for the condition of *mdx* mice and represents a new therapeutic scenario for fibrotic muscle diseases [149,150]. Similarly, augmenting integrin β1 signaling with an anti-Fgf2 antibody greatly improved satellite cell regenerative function and enhanced muscle regeneration in *mdx* mice [151]. The study by Rozo and colleagues [151] is an excellent example of combining outstanding basic science with a treatment method.

The many other strategies that explore downstream disease mechanisms from different angles and tackle different processes (especially in *mdx* mice) cannot be fully described in this article (a detailed review of pharmacological treatment for dystrophin-deficiency is presented in [29]). However, the studies discussed here indicate that single-mode therapies might be insufficient to combat the multi-faceted pathology of MD [127].

#### Genetic Modifiers 

The progression of the dystrophic condition and extensive variability of clinical phenotypes has been attributed not only to primary genetic mutations. Secondary mutations, gene polymorphisms, and differential expression levels of a wide array of genes account for inter-individual variability in patients and differences among strains in laboratory mice [152]. The genes affected by secondary variations are called genetic modifiers and they have recently gained attention in the context of novel drug development, as they could provide a platform for identification of novel pharmacological targets or pathways to counteract dystrophic progression. For example, LTBP4, osteopontin and Jagged 1 have been found to be genetic modifiers in *mdx* mice, dystrophin-deficient dystrophic dogs, and human patients [153,154,155,156], regulating the disease progression by interference with pro-fibrotic and pro-regenerative pathways (TGF-β, myostatin and Notch signaling) [152,156]. Additionally, increased components of polyamine pathway metabolism (Amd1, Smox) have been shown to lessen the severity of triceps muscle condition in mice bearing laminin α2 chain mutation (*dy^2J^/dy^2J^* mice) [157]. 

Thrombospondin-4, well known for its role in the extracellular compartment, could be a genetic modifier in deficiencies involving the dystrophin-glycoprotein complex, as increased expression levels of thrombospondin-4 have been shown to have a protecting effect and promote skeletal muscle integrity in mouse models for δ-sarcoglycan and dystrophin deficiency. Interestingly, stabilization of muscle membrane in dystrophic mice overexpressing thrombospondin-4 was achieved through direct interaction of intracellular fraction of thrombospondin-4 with activating transcription factor 6 (ATF6α). This interaction triggered the enhanced vesicular trafficking between endoplasmic reticulum, Golgi apparatus, and sarcolemma, and augmented endoplasmic reticulum stress adaptation [158]. 

Taken together, various molecular pathways provide unique opportunities for the development of novel medicinal products to combat muscle degeneration and fibrosis, so the field of genetic modifiers is also moving the translational opportunities forward [152]. 

## 4. Clinical Trials

The availability of adequate animal models, extensive studies of disease pathogenesis, and development of new treatment concepts have established a solid framework for therapeutic applications in clinical trials. Over 200 clinical trials regarding MD have been registered in the US and over 60 in Europe (www.clinicaltrials.gov; www.clinicaltrialsregister.eu) (including both open and closed studies). Most of those trials concern patients with Duchenne/Becker MD (reviewed in [3]). Tests for limb-girdle, fascioscapulohumeral, and congenital MDs have made a long-awaited appearance in trial registers and are becoming more frequent. However, many MD treatment trials still focus on symptom alleviation, not cure. 

It cannot be ignored that a few medications yielded very promising results in mice, and even dogs, but the tests with human subjects were less convincing (ataluren, eteplirsen and other exon skipping agents). Nevertheless, there is an encouraging growing interest in clinical trials for MD, even for more rare forms. Numerous small companies have been set up by investors and researchers who have published promising results. These companies pursue the final evaluation of efficacy and safety of treatments in animal models with a primary goal to prepare and finally enter the clinical phase of research. 

Development of clinical trials for orphan disorders will always suffer from various logistic and financial limitations. However, the refinement of legislative processes and international cooperation between industry, scientists, clinicians, and administrative bodies is a step in the right direction that may open new avenues for the translational opportunities. 

This review has emphasized that the understanding of genetic and pathogenic mechanisms of the disease, although greatly advanced in recent decades, has not resulted in outstanding increase of cure implementation. It has become even clearer that further development of genome-related and pharmaceutical technologies, in combination with basic science and preclinical studies, is urgently needed for successful clinical trials. It is particularly important to carefully consider new trials, especially when the number of patients with rare disorders is limited. Extraordinary measures should be taken to avoid unnecessary exposure of patients to a burden of going through exhausting procedures, but also to prevent keeping patients out of tests for therapies that may have a better chance of success.

### Animal Models Versus Clinical Trials—Alternative Preclinical Research

Although the research on animal models has taken us so far, it cannot be overlooked that over 80% of treatments successfully tested on animals have failed in clinical trials [159]. The MD field has also suffered in that respect: generally low numbers of translational attempts, and a low success rate of clinical studies (as well as lack of a ‘spectacular’ outcome) in the MD field is not an exception. The crisis between preclinical and translational science has become a fact, and it hinders the development of new clinical tests. We must bear in mind that successful completion of clinical trials and new drug development is associated with enormous financial cost, complicated and long procedures and, most importantly, human costs (commitment to trial regime, unwanted side effects, potential disappointment).

Why are the results obtained in animal models ‘lost in translation’? There are several reasons. The design of preclinical experiments is often not sufficiently rigorous (lack of proper control groups, small number of animals used, insufficient attention to pharmacodynamics and pharmacokinetics of drugs, bias, incomplete presentation of relevant data, and negligence of negative results). Unfortunately, the pressure to publish is very strong in academia, pushing investigators to ignore the fact that their results may be premature and not detailed enough. Additionally, standard operating protocols that would require all research groups to use a certain mouse model to follow the exact same protocols are still discussed and remain at an early stage of development. All of this leads to frequent irreproducibility of scientific data [160,161]. 

Another factor contributing to implementation limitations lies in the general differences in genetics, physiology, and behind variations in the homology of specific molecular targets between mice and humans [162]. Weaknesses in faithfully mirroring the extremely complex pathological processes in humans are difficult to overcome using animal models, especially if only a single inbred mouse strain is available to model a disease. Is research on animals, therefore, overexaggerated, overused, and obsolete? The extent of research on animals could be debatable [163,164], but it is difficult to completely replace animal models. It cannot be denied that research on mice, rats, and other species has facilitated great strides in understanding various diseases, including MD. Animal models still offer opportunities for the clinical sector and valuable knowledge to support development of treatments. Accordingly, techniques established through preclinical studies with animals have advanced to clinical trials with MD patients. Some of those techniques yielded relatively optimistic results and have taken us one step closer to defeating the disease. Nevertheless, a few aspects need to be considered when predicting clinical efficacy based on animal research: mouse models used in a preclinical setting with the aim of progressing the treatment to clinical trials need to be completely characterized, especially regarding signaling pathways, regulatory mechanisms, and genetic factors that could influence the targeted pathogenic mechanism. Factors creating noise in data that could lead to spurious conclusions must be excluded [159]. 

A better understanding of human pathologies should go hand in hand with the characterization of animal models. The complexity of pathogenic mechanisms in humans is further exacerbated by individual differences between patients, which are difficult to control. This has also shown to be an obstacle when using large animals in preclinical set-ups (e.g., dogs) [64,155]. There is much more scope for optimizing procedures when working with a mouse strain that shows relatively little variability between individuals. 

Failure of a clinical trial could, therefore, be associated with multiple factors, including insufficient knowledge about the human condition and weaknesses of a clinical trial design. It cannot be excluded that custom drugs adjusted for each patient according to the individual disease characteristics will need to be considered in the future.

A wide range of alternatives to animal-based preclinical research are available that could facilitate clinical trials. These alternatives include well-known classical approaches, such as epidemiological studies and in vitro human cell-based assays, which continue to be optimized and improved. New methods are emerging in biomedical research that could open new avenues to efficient design of clinical trials. For example, ‘human organs on a chip’ and ‘microfluidic chips’ that create living systems by mimicking a microbiological environment with cells of a certain organ implanted onto silicon chips are considered to become a future of preclinical research. Additionally, in silico computer modelling developed to model pharmacologic or physiologic processes using explosive increases of computing power could also become a future preclinical-clinical link/axis [162,163].

## 5. Concluding Remarks

Maickel Melamed, a 41-year-old muscular dystrophy patient, has not let the disease stop him from achieving his dreams. He completed the New York, Tokyo, Chicago, Berlin and, finally, the Boston marathons. This last one took him 20 grueling hours to reach the finish line, against the odds and overcoming obstacles of constant pain and pouring rain. Despite this, Maickel never gave up, having fans and friends cheering for him. His attitude, his long and challenging journey is a tribute to all muscular dystrophy patients, but also an enormous inspiration to physicians and researchers. 

Our work to combat muscular dystrophy continues to be demanding and results are eagerly awaited. Even a small improvement in a patient’s condition means the world to them. However, it does not end here—we can defy the odds and overcome this dramatic disorder. Clinicians, researchers, investors, and governing and legislative bodies need to join forces to help us reach this goal. Creativity in research is rapidly extending the limits and setting new standards for treatment design. People like Maickel motivate us to work even harder towards full understanding of muscular dystrophy pathology and to persistently pursue a cure for the disease. I strongly believe that this mission will be achieved in the future. 

## Figures and Tables

**Table 1:**
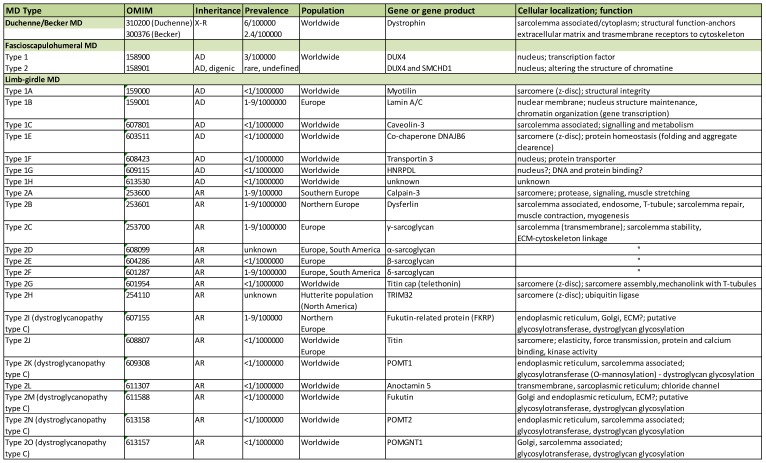

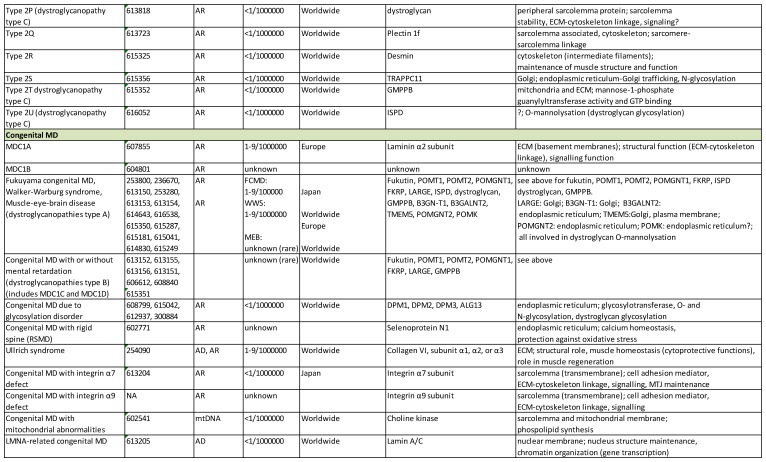

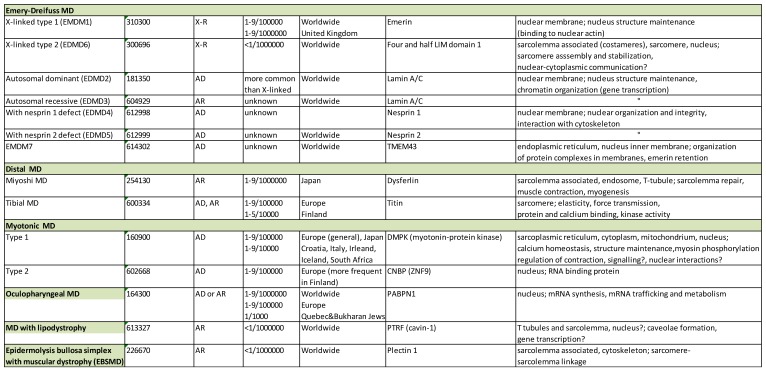
MD classification has become increasingly complex. The classification and gene information presented here is based on the Online Mendelian Inheritance in Man (OMIM) database (http://omim.org), the GeneCards database (www.genecards.org), the MalaCards human disease database (www.malacards.org), the Orphanet epidemiological database (www.orpha.net) and the Neuromuscular Disorders Journal list of muscle diseases. AR: autosomal recessive; AD: autosomal dominant; X-R: X-linked; ECM: extracellular matrix; MTJ: myotendinous junction; WWS: Walker-Warburg syndrome; MEB: muscle-eye-brain disease; ?: putative function.

**Table 2:**
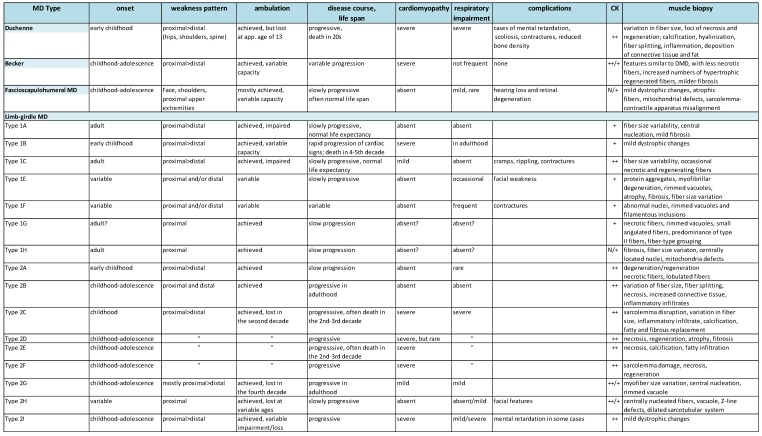

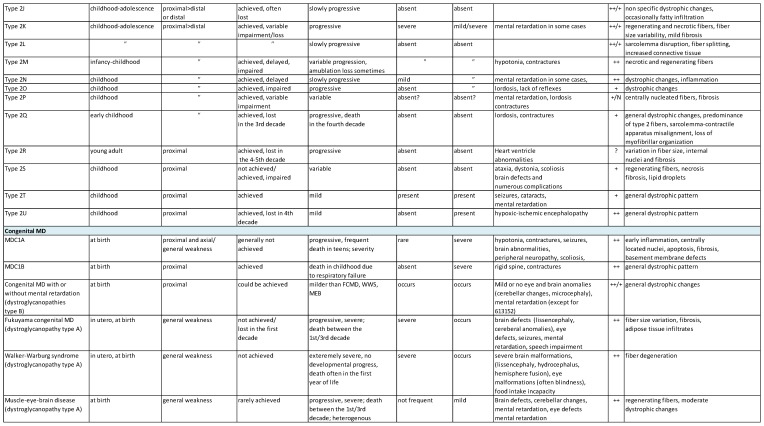

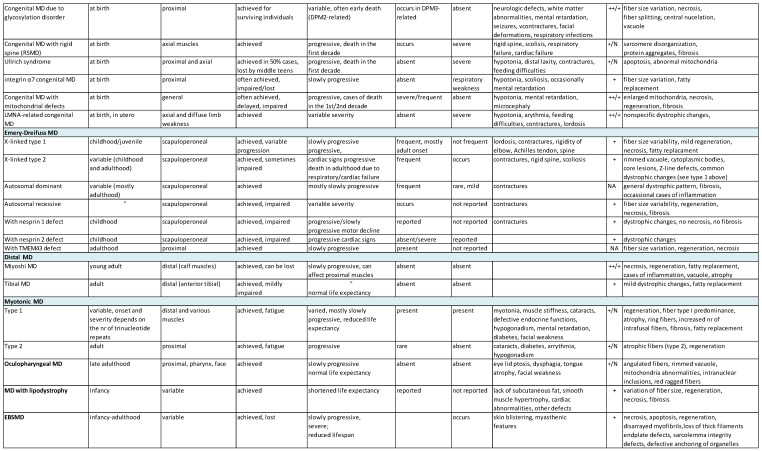
Spectrum of MD clinical features. Based on: [1,4]; the Online Mendelian Inheritance in Man (OMIM) database (http://omim.org), the Orphanet epidemiological database (www.orpha.net), Gene Reviews^®^ [Internet], ([5] https://www.ncbi.nlm.nih.gov/books/NBK1116/?term=gene%20reviews). CK: creatine kinase; CNS: central nervous system; ++: substantially increased; +: increased; N: normal; NA: not applicable.

**Table 3:**
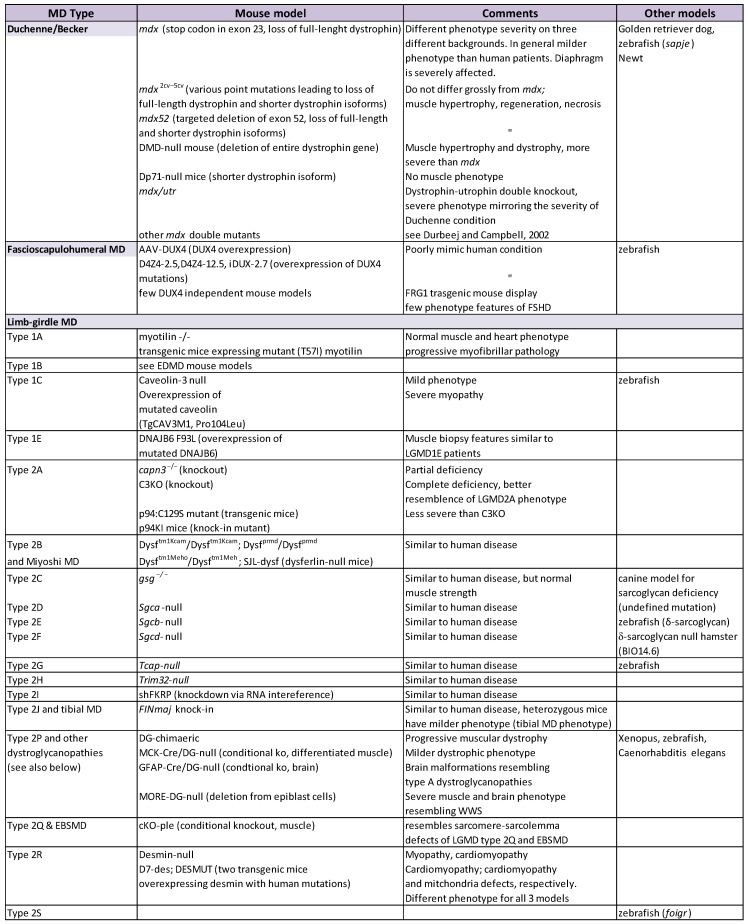

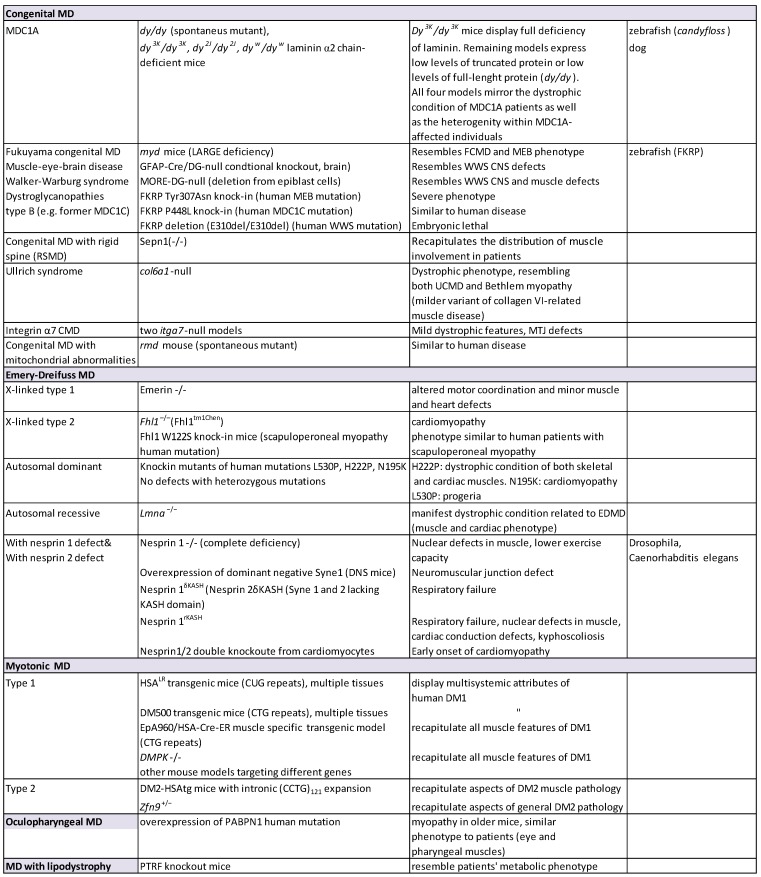
Animal models for MD. Based on [23,24]; Mouse Genome Informatics Database http://www.informatics.jax.org/. CNS: central nervous system; ko: knockout; WWS: Walker-Warburg syndrome; MEB: muscle-eye-brain disease; MTJ: myotendinous junction.
